# Primary prevention ICD in non-ischaemic cardiomyopathy: an ongoing search for improvement of current indications: A retrospective study analysing the impact of the new Dutch guideline on the use of ICDs

**DOI:** 10.1007/s12471-025-01960-5

**Published:** 2025-05-12

**Authors:** Reinder Evertz, Rypko Beukema, Sjoerd Westra, Robin Nijveldt, Kevin Vernooy

**Affiliations:** 1 Department of Cardiology, Radboud University Medical Centre, Nijmegen, The Netherlands; 2 Deparment of Cardiology, Cardiovascular Research Institute Maastricht, Maastricht University Medical Centre, Maastricht, The Netherlands

**Keywords:** Implantable cardioverter-defibrillator, Non-ischaemic cardiomyopathy, Dutch guidelines, Sudden cardiac death risk

## Abstract

**Introduction:**

Patients with non-ischaemic cardiomyopathy (NICMP) have a class IIa primary prevention indication for an implantable cardioverter-defibrillator (ICD). Recent studies have shown that the evidence for a survival benefit following ICD implantation in this patient group is not particularly robust. In 2023, the Dutch Society of Cardiology published an update of the ESC guideline to better select patients with NICMP for ICD implantation. The objective of this study was to analyse the impact of this guideline on the number of indications in a retrospective cohort of patients who had received an ICD and whether the patients without an indication were also without appropriate ICD therapy.

**Methods:**

A single-centre, retrospective observational study was performed in 134 patients with NICMP who underwent ICD implantation for primary prevention between 2015 and 2020.

***Results*:**

After applying the new Dutch guideline, 74 out of 134 patients with NICMP without a high-risk phenotype (35 patients) had no ICD indication (group 2). The remaining 25 patients were considered to have an ICD indication (group 1). During a median follow-up of 66 months (interquartile range 52–81) the incidence of appropriate ICD therapy (antitachycardia pacing and shock) was comparable in both groups: 4 patients in group 1 (16%) and 9 in group 2 (12%), *p* = 0.623.

***Conclusion*:**

The new 2023 guideline for ICD implantation in NICMP patients does indeed rule out a significant group of patients from ICD implantation. Nevertheless, our data show that patients without an indication still had comparable rates of appropriate ICD therapy.

## What’s new?

- This is the first retrospective analysis of the impact of the 2023 updated Dutch guideline for implantable cardioverter-defibrillator (ICD) therapy for primary prevention in patients with non-ischaemic cardiomyopathy.
- Although this guideline does significantly lower the number of patients with an indication for an ICD, these patients are not necessarily without risk of ventricular arrhythmias.

## Introduction

Patients with heart failure with reduced systolic left ventricular function have an increased risk of mortality. Current European guidelines recommend an implantable cardioverter-defibrillator (ICD) in symptomatic patients with a left ventricular ejection fraction (LVEF) ≤ 35%, despite optimal medical therapy [1, 2]. Several clinical studies have shown a survival benefit in patients with ischaemic heart disease and a reduced LVEF, but in non-ischaemic cardiomyopathy (NICMP) patients the evidence is less robust [3–7]. The DANISH trial showed a relatively low risk of sudden cardiac death (SCD) compared to previously performed studies and adding an ICD to cardiac resynchronisation therapy (CRT) did not improve survival [6]. Nevertheless, meta-analyses on the use of ICDs in NICMP patients are still in favour of ICD, even without a CRT indication [8]. In recent years, medical treatment of heart failure has evolved with improved overall survival [9–12]. Several studies have shown the relationship between the presence of late gadolinium enhancement (LGE) on cardiac magnetic resonance (CMR) and a higher risk of ventricular arrhythmias, SCD and overall survival [13–18]. Given these results and the difficulty in predicting the risk of SCD in NICMP, at the instruction of the National Health Care Institute the Netherlands Society of Cardiology recently published an updated indication guideline. The use of CMR, the use of a competing risk model for mortality and genetic testing is implemented, and an estimated 70% reduction of ICD implantations in this patient group is expected [19–21].

**Fig. 1:**
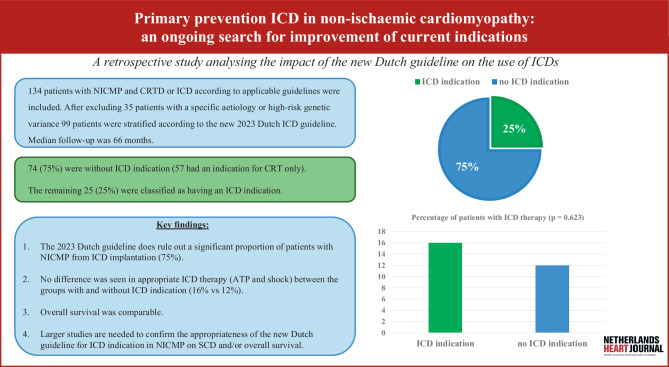
Infographic: primary prevention implantable cardioverter-defibrillator (*ICD*) in non-ischaemic cardiomyopathy (*NICMP*). *CRT* cardiac resynchronisation therapy, *CRTD* cardiac resynchronisation therapy with a defibrillator, *ATP* antitachycardia pacing, *SCD* sudden cardiac death

The purpose of this exploratory study was to evaluate the effect of this new Dutch guideline on a patient cohort with a previously implanted ICD and/or cardiac resynchronisation therapy with a defibrillator (CRTD) at the Radboud University Medical Centre (Radboudumc), Nijmegen, The Netherlands (Fig. 1: Infographic).

## Methods

### Study population

This retrospective, observational study enrolled 99 patients with NICMP who underwent ICD implantation for primary prevention between 2015 and 2020 at the Radboudumc. An ICD for primary prevention was implanted according to the guidelines in force at the time (mainly defined as symptomatic heart failure and LVEF of ≤ 35% despite optimal medical therapy). Patients younger than 18 years at the time of implantation or patients who had stated that they did not want their data used for scientific research were excluded.

### Methods

Data were obtained from the patients’ medical records and device interrogation and analysed retrospectively. Analysis of the device interrogation was performed in order to characterise the type of therapy (antitachycardia pacing (ATP), ICD shock, or both), whether it was appropriate or inappropriate. The relevant episode data, such as duration of the arrhythmia, time to diagnosis and therapy type, were reviewed for each episode. The revision and assessment of the stored ICD therapy was done by a qualified electrophysiologist. The study was approved by the local ethics committee (file number 2021-8215).

### The 2023 Dutch guideline

On the instruction of the National Health Care institute, the Netherlands Society of Cardiology published a new guideline for ICD indication in NICMP. The society emphasises the use of the ESC guidelines but made additional recommendations based on results from recently published studies. An effort to better diagnose the aetiology of the cardiomyopathy is emphasised by a class I indication to perform CMR with LGE and to perform genetic testing. In an effort to better define life expectancy as given in the statement of the ESC guidelines that an ICD should be considered only in patients with an expectation of good quality of life > 1 year, a validated survival risk score (the heart failure meta-score) was added with the recommendation not to implant an ICD in patients with a 1-year-mortality risk score > 35% [19].

### Study outcomes

The primary outcome of the study was the number of patients with and without an indication for primary prevention ICD in NICMP according to the new Dutch guideline and the incidence of appropriate ICD therapy, consisting of both ATP and shock for ventricular arrhythmias in these two groups. Secondary outcomes consisted of the incidence of inappropriate ICD therapy, device-related complications and overall mortality.

### Statistical analysis

In this study, categorical or continuous variables were used. Categorical data were presented as absolute numbers and percentages. Continuous variables were expressed as mean ± SD or median (interquartile range (IQR) Q1–Q3) for skewed distributions. Differences at baseline were compared for patients with and without an ICD indication. These data were was analysed using a Student’s *t*-test or Mann-Whitney test for the numerical variables and a chi-square test or Fisher’s exact test for the categorical variables. The secondary outcomes were also analysed with a chi-square or Fisher’s exact test. Mortality rate was compared between groups with the Kaplan-Meier method and the log rank test. The statistical analyses were performed in SPSS Statistics version 29 (IBM Corp., Armonk, NY, USA). All the *p*-values were two-sided, and a *p*-value < 0.05 was considered to be statistically significant.

## Results

### Baseline characteristics

**Fig. 2:**
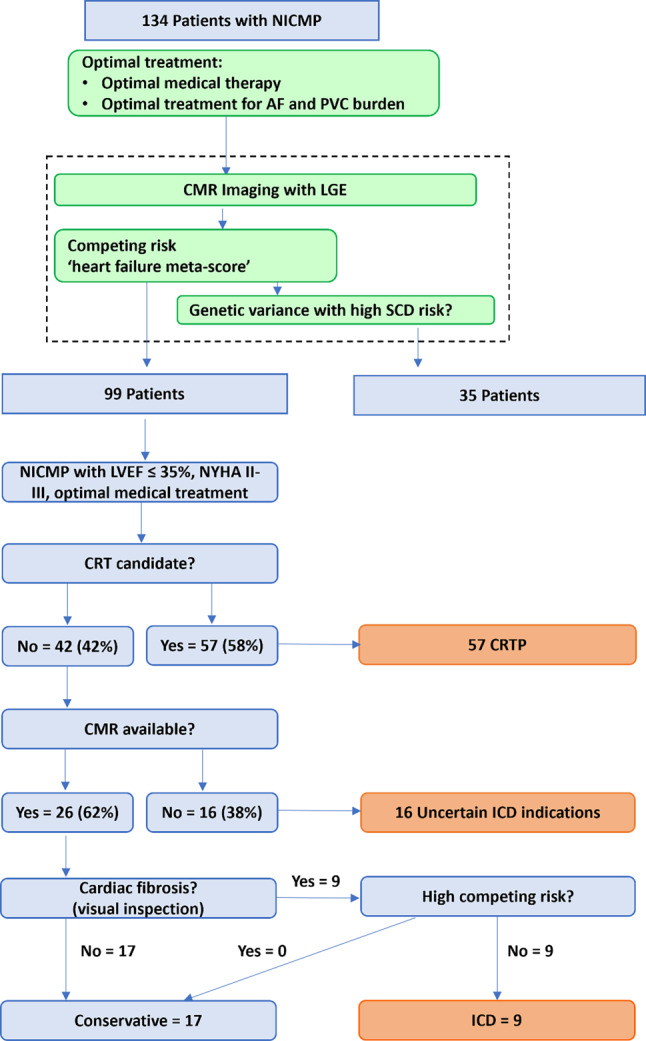
Flow chart of the 2023 Dutch guideline for the indication for implantable cardioverter-defibrillator (*ICD*) therapy in non-ischaemic cardiomyopathy (*NICMP*). Ninety-nine patients out of 134 with NICMP were included. A description of the heart failure meta-score can be found at: http://www.hfmetascore.org. *AF* atrial fibrillation, *PVC* premature ventricular contraction, *CMR* cardiac magnetic resonance, *LGE* late gadolinium enhancement, *SCD* sudden cardiac death, *LVEF* left ventricular ejection fraction, *NYHA* New York Heart Association class, *CRT* cardiac resynchronisation therapy, *CRTP* CRT pacing

Between 2015 and 2020 a total of 134 patients with NICMP received an ICD for primary prevention at the Radboudumc. Thirty-five patients had a specific aetiology for their cardiomyopathy or a high-risk genetic variant (18 hypertrophic cardiomyopathy, 2 arrhythmogenic right ventricular cardiomyopathy, 3 cardiac sarcoidosis and 12 high-risk genetic variants (4 PLN, 1 RBM-20, 2 DSP and 5 LMNA)). The remaining 99 patients were then analysed according to the flow chart of the 2023 Dutch guideline for ICD indication (Fig. 2). Tab. 1 shows the baseline characteristics. Clinically, 55 patients with NICMP received CRTD and 44 an ICD.

**Table 1:** Baseline characteristics. Continuous variables are presented as mean ± SD or median (IQR) for skewed distributions, and categorical variables are presented as numbers (%)

	All	Group 1: ICD indication	Group 2: no ICD indication	*p*-value
Total number	99	25 (25%)	74 (75%)	
ICD indication according to 2023 Dutch guideline		9 clear, 16 uncertain	57 CRTP, 17 no ICD	
CRTD	55 (56%)			
ICD (single and dual chamber)	44 (44%)			
Male gender	61 (62%)	17 (68%)	44 (60%)	0.447
Age at implantation (years), mean (SD)	64 (11.5)	61 (11)	65 (11.5)	0.176
BMI (kg/m^2^), mean (SD)	29 (5.5)	28.4 (4.8)	29 (5.7)	0.488
LVEF (%), mean (SD)	27 (6.2)	28 (6.6)	26 (6)	0.145
*NYHA functional class*				
NYHA I	9 (9%)	3 (12%)	6 (8%)	0.6
NYHA II	68 (69%)	18 (72%)	50 (68%)	0.68
NYHA III	22 (22%)	4 (16%)	18 (24%)	0.36
QRS duration (ms), mean (SD)	139 (32)	107 (14)	150 (30)	< 0.001
eGFR (ml/min/1.73 m^2^), median (IQR)	64.8 (53–79)	70 (55–90)	63.5 (50–79)	0.386
Atrial fibrillation	27 (27%)	11 (44%)	16 (22%)	0.054
Diabetes mellitus	22 (22%)	6 (24%)	16 (22%)	0.813
COPD	10 (10%)	4 (16%)	6 (8%)	0.339
Cerebrovascular disease	15 (15%)	1 (4%)	14 (19%)	0.016
CMR available	59 (60%)	9 (36%)	50 (68%)	0.008
LGE on CMR	19 (32% of 59)	9 (100%)	10 (20%)	0.002
Heart failure meta-score > 35% 1‑year mortality	0 (0%)	0 (0%)	0 (0%)	
Follow-up (months), median (IQR)	66 (52–81)	70 (40–81)	66 (55–81)	0.587

*ICD* implantable cardioverter-defibrillator, *CRTD* cardiac resynchronisation therapy defibrillator, *BMI* body mass index, *LVEF* left ventricular ejection fraction, *NYHA* New York Heart Association class, *eGFR* estimated glomerular filtration rate, *COPD* chronic obstructive pulmonary disease, *CMR* cardiac magnetic resonance, *LGE* late gadolinium enhancement

### Stratification according to the new Dutch guideline

According to the flow chart of the 2023 Dutch guideline 57 of the 99 patients had an indication for CRT only. In 26 (62%) of the remaining 42 patients, CMR was available, so in 16 patients it was not clear whether there was an ICD indication. Out of the 26 patients with CMR, 9 had visually determined LGE as proof of fibrosis and could therefore be classed as having an ICD indication. The heart failure meta-score of these patients and all others was below 35% (Tab. 1). Thus 9 out of the 99 patients (9%) had an ICD indication according to the new Dutch guideline and 74 did not (57 CRT pacing (CRTP) and 17 without LGE on CMR). CMR was not available in 16 patients, but LGE would probably have been detected on CMR in some of these cases. Thus, in order not to overestimate the number of patients without an ICD indication, all 16 patients without CMR were considered to have an ICD indication and were therefore added to the ones with a clear indication, making a total of 25 patients in group 1 (ICD indicated). Group 2 (no ICD indicated) consisted of the 74 patients without an indication.

### ICD therapy

During a median follow-up of 66 months (IQR 52–81) 13 of the 99 patients received appropriate ICD therapy (either ATP or shock), 4 (16%) in group 1 and 9 in group 2 (12%) (*p* = 0.623). This represents an annual event rate of 2.4% in the total cohort. Three patients in group 1 and 5 patients in group 2 received an ICD shock. CMR was not available in the 4 patients in group 1 (Tab. 2).

**Table 2:** Follow-up. Categorical data are presented as numbers (%). Patients selected according to 2023 Dutch guideline with or without an indication for implantable cardioverter-defibrillator (*ICD*) therapy

	Group 1: ICD indication	Group 2: no ICD indication	*p*-value
Total number	25 (25%)	74 (75%)	
Appropriate ICD therapy (ATP and shock)	4 (16%)	9 (12%)	0.623
– ATP	1	4	
– Shock	3	5	
– CMR+	0/9		
– CMR−	4/16 (25%)		
Inappropriate ICD therapy (ATP and shock)	4 (16%)	4 (5%)	0.093
Device-related problems (lead-related/infection/pocket)	2 (1%)	7 (10%)	0.826
– Lead-related	0	6	
– Infection	2	0	
– Pocket	0	1	
Deceased during follow-up	9 (36%)	13 (18%)	0.055

*ATP* antitachycardia pacing, *CMR* cardiac magnetic resonance

### Inappropriate ICD therapy and complications

A total of 8 patients (8%) had inappropriate ICD therapy during follow-up (4 (16%) in group 1 and 4 (5%) in group 2, *p* = 0.093). In 7 patients inappropriate ICD therapy was due to a supraventricular arrhythmia, mainly atrial fibrillation, and in one due to noise sensing as a result of lead failure. Nine patients (9%) in the total cohort had a complication related to the pocket, device or leads: 2 patients in group 1 (1%) and 7 in group 2.

### Survival

**Fig. 3:**
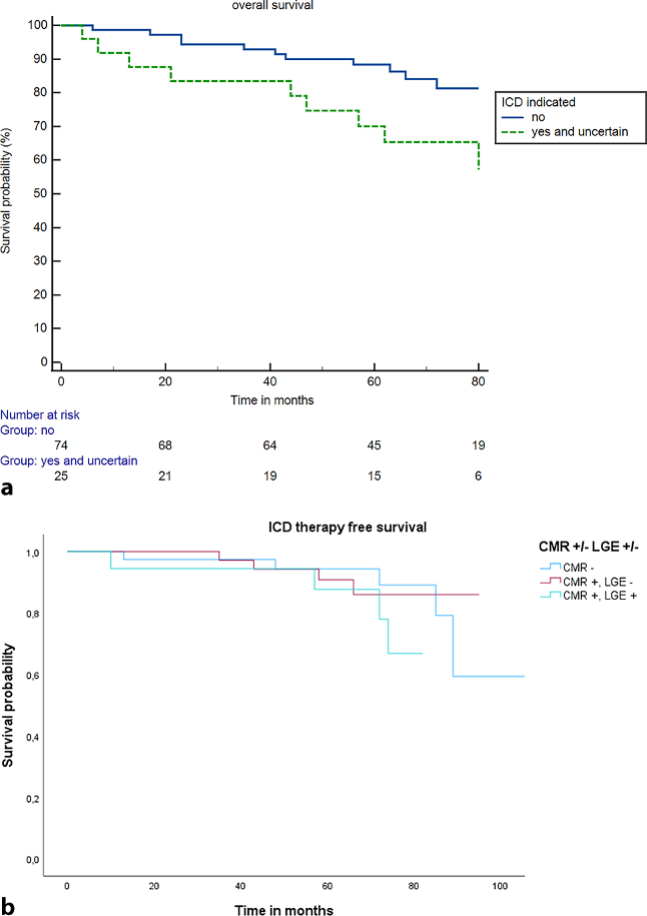
**a** Kaplan-Meier curve for overall survival. Mortality rate in the patients with and without an indication for implantable cardioverter-defibrillator (*ICD*) therapy. Log rank = 0.055. **b** Kaplan-Meier curve for ICD therapy-free survival: Patients selected according to the availability of cardiac magnetic resonance (*CMR+* or *CMR−*) with or without late gadolinium enhancement (*LGE+* or *LGE−*)

Twenty-two patients (22%) died during follow-up due to end-stage heart failure or non-cardiac causes. There was no significant difference in overall survival between the two groups: 9 patients in group 1 (36%) compared to 13 patients in group 2 (18%), *p* = 0.055. Only one patient died due to SCD with ongoing ventricular fibrillation after an ICD shock. This patient had no ICD indication (group 2). Figure 3a shows the Kaplan-Meier survival curve of these two groups.

### ICD therapy according to the availability of CMR and LGE

In 40 out of 59 patients with CMR, LGE was available, whereas in 19 it was not. Appropriate ICD therapy was seen in 21% in the group for which LGE was visible and 10% in the group where it was not (*p* = 0.247). In the patient group in which CMR was not performed, the incidence of appropriate ICD therapy was 13% (Tab. 3; Fig. 3b).

**Table 3:** Follow-up. Categorical data are presented as numbers (%). Patients selected according to the availability of cardiac magnetic resonance (*CMR+* or *CMR−*) with or without late gadolinium enhancement (*LGE+* or *LGE−*)

	CMR+, LGE−	CMR+, LGE+	CMR−	*p*-value (LGE+ vs LGE−)
Total number	40	19	40	
Appropriate ICD therapy (ATP and shock)	4 (10%)	4 (21%)	5 (13%)	0.247
– ATP	2	1	3	
– Shock	2	3	2	

*ICD* implantable cardioverter-defibrillator, *ATP* antitachycardia pacing

## Discussion

Our study shows that the percentage of patients with NICMP without an indication for ICD therapy according to the new Dutch guideline was indeed high: excluding the high-risk patient group, 74 patients out of 99 (75%) would not have had an ICD implanted, mainly because of a CRTP indication (57 out of 74, 77%). This new guideline therefore does indeed reduce significantly the number of ICD indications in this specific patient group. Nevertheless, and importantly, appropriate ICD therapy (both ATP and shocks) had a comparable incidence of 16% in the group with an indication versus 12% in the group without. The estimated annual risk for appropriate ICD therapy for ventricular arrhythmias of 2.4% is comparable with the incidence mentioned in the literature of between 2 and 4% in ICD for primary prevention (ICMP and NICMP patients, respectively) [22–24].

One of the main parameters in the 2023 Dutch guideline for ICD indication is the presence of fibrosis (or LGE) on CMR. In our single-centre retrospective study, of the group with an ICD indication according to the 2023 Dutch guideline 16 patients did not undergo CMR, so were without a clear indication. Because of the exploratory, retrospective nature of the study these patients were not excluded from the analysis so as not to underestimate the event rate in an already limited sample size. Interestingly the 9 with a clear indication because of LGE on CMR did not have ICD therapy during follow-up, but of the 16 without CMR, 4 patients did. In the total population studied, CMR was performed in 59 out of the 99 patients. Appropriate ICD therapy was seen in patients without CMR, in patients with LGE on CMR and even in patients without LGE on CMR. Our data could not confirm that the absence of LGE completely ruled out the risk of ventricular arrhythmias. Interobserver variability in the interpretation of the presence of LGE on CMR has to be close to zero using the sole criteria of presence of absence of LGE for an ICD indication.

Overtreating patients implies unnecessary exposure to the risk of complications, with a published complication rate of 1 out of 13 patients after ICD implantation [25]. Our data confirm this complication rate with 9% complications in the total group, mainly related to lead problems. Inappropriate ICD therapy was comparable in both groups and was mainly driven by supraventricular arrhythmias.

Improvements in treatment over the past years have significantly impacted the lives of heart failure patients as regards both prognosis and quality of life, but we are still not able to properly select patients who are at low risk for SCD. Our study results question the fact that the new Dutch guideline could completely or sufficiently rule out the risk of life-threatening ventricular arrhythmias in patients with NICMP, since the event rate of appropriate ICD therapy was comparable for patients with. and without an ICD indication. A better understanding of the mechanism of ventricular arrhythmias is needed in this population. More data are needed to understand and redefine ICD indications in this specific patient group with NICMP. A multicentre study to gather more retrospective data to enlarge the sample size could add valuable insights into the impact of the new Dutch guideline. The next step could be a prospective, randomised study to show the benefit and capability of the new Dutch guideline to properly exclude patients from ICD implantation. With an estimated and published annual risk of around 4% of ventricular arrythmias in patients with an ICD for primary prevention, a comparison to an acceptably low annual SCD risk should be made [22–24]. There is no consensus about this accepted annual risk, but 1% has been discussed. In a non-inferiority design and with the established 4% versus 1% annual SCD risk rate, with 80% power and 5% type I error, 424 patients per group would be needed in such a study. Several ongoing or soon to be started studies on ICD therapy, SCD risk and NICMP, such as the BRITISH, the CONTEMP-ICD and Dutch-ICD study will most likely show the beneficial effect of ICD on survival and provide valuable insights into specific clinical risk factors to further redefine ICD indication in this difficult patient group.

A recently published position paper dealt with risk assessment and ICD indication in NICMP. A more individualised approach was recommended, weighing arrhythmic risk, competing risks and possible risk modifiers to assess eligibility. The same steps, including CMR, genetic testing and ‘competing’ risk scores, as proposed in the 2023 Dutch guideline, were used. ICD therapy was not completely ruled out in patients eligible for CRTP. However, the authors concluded that probably only a small proportion should be considered for CRTD (with a probably high SCD risk) and that in the group of patients with NICMP without an indication for CRTP, a subgroup at high risk should have an ICD implanted. We are obligated to refine the SCD risk and define the patient at truly low risk [26]. The position paper puts current knowledge into perspective and warrants further research once again.

### Limitations

The retrospective study design is a limitation and lacks sufficient statistical power. ICD therapy could have been underestimated, since missing data is a concern in retrospective analysis. Since treatment strategies and ICD programming have changed over time, this could influence outcome when applied correctly.

## Conclusion

The new 2023 Dutch guideline as an addendum to the currently available ESC guideline for ICD implantation in NICMP patients is an attempt at refining the ICD indication criteria in patients with NICMP, but it did not sufficiently predict the risk of ICD therapy in our cohort. Further research is warranted, while in the meantime an individualised approach has to be emphasised.
